# *Clostridium difficile* in Discharged Inpatients, Germany

**DOI:** 10.3201/eid1301.060611

**Published:** 2007-01

**Authors:** Ralf-Peter Vonberg, Frank Schwab, Petra Gastmeier

**Affiliations:** * Medical School Hannover, Hannover, Germany; † Charité – University Medicine Berlin, Berlin, Germany.

**Keywords:** Clostridium difficile, epidemiology, Germany, letter

**To the Editor:** Using discharge diagnoses from US hospitals in 2000–2003, McDonald et al. recently documented a dramatic increase in the rate of *Clostridium difficile*–associated disease (CDAD) (*1*). During the same period, a new strain of *C. difficile* was identified; this strain appears more virulent, at least in part because it produces higher levels of toxin (*2*).

To our knowledge, this strain has not been identified in Germany. However, to address this emerging threat, we conducted a similar analysis of discharge data to compare findings from the United States with data from Germany. We therefore determined the absolute number of inpatient discharges from all hospitals in Germany with the number of discharge diagnoses of CDAD reported in the national Statistische Bundesamt for the years 2000–2004. We then calculated the incidence of CDAD as a discharge diagnosis for each year and stratified our results by age groups (Figure).

**Figure:**
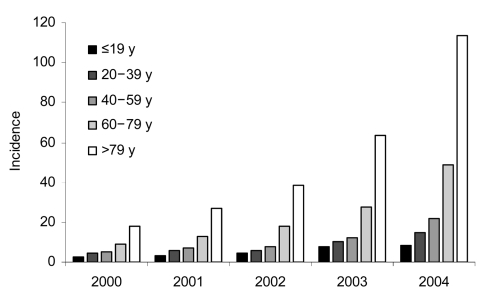
Incidence of *Clostridium difficile*–associated disease per 100,000 inpatients upon discharge from hospitals in Germany.

Our results confirm the observations from the United States. The effect of *C. difficile* on illness of patients in hospitals in Germany has escalated dramatically. This is true especially for patients >60 years of age. This trend indicates the need for increased awareness of this pathogen and a concerted effort to control CDAD by reducing unnecessary antimicrobial drug use and implementing currently recommended infection control measures. It also highlights the need to develop more rapid and accurate diagnostic tools and more effective prevention and treatment strategies.
